# Renal unit practitioners’ knowledge, attitudes and practice regarding the safety of unfractionated heparin for chronic haemodialysis

**DOI:** 10.4102/curationis.v38i1.1447

**Published:** 2015-09-16

**Authors:** Debra Ockhuis, Una Kyriacos

**Affiliations:** 1 Faculty of Health Sciences, Department of Health and Rehabilitation Sciences, University of Cape Town, South Africa

## Abstract

**Background:**

Chronic haemodialysis for adult patients with end-stage kidney failure requires a patent extracorporeal circuit, maintained by anticoagulants such as unfractionated heparin (UFH). Incorrect administration of UFH has safety implications for patients.

**Objectives:**

Firstly, to describe renal practitioners’ self-reported knowledge, attitudes and practice (KAP) regarding the safe use of UFH and its effects; secondly, to determine an association between KAP and selected independent variables.

**Method:**

A cross-sectional descriptive survey by self-administered questionnaire and non-probability convenience sampling was conducted in two tertiary hospital dialysis units and five private dialysis units in 2013.

**Results:**

The mean age of 74/77 respondents (96.1%), was 41.1 years. Most (41/77, 53.2%) had 0–5 years of renal experience. The odds of enrolled nurses having poorer knowledge of UFH than registered nurses were 18.7 times higher at a 95% Confidence Interval (CI) (1.9–187.4) and statistically significant (*P* = 0.013). The odds of delivering poor practice having ≤ five years of experience and no in-service education were 4.6 times higher at a 95% CI (1.4–15.6), than for respondents who had ≥ six years of experience (*P* = 0.014) and 4.3 times higher (95% CI 1.1–16.5) than for respondents who received in-service education (*P* = 0.032), the difference reaching statistical significance in both cases.

**Conclusion:**

Results suggest that the category of the professional influences knowledge and, thus, safe use of UFH, and that there is a direct relationship between years of experience and quality of haemodialysis practice and between having in-service education and quality of practice.

## Introduction

### Problem statement

Patients with end-stage kidney failure (ESKF) depend on safe chronic haemodialysis therapy to restore homeostasis. Safe haemodialysis treatment includes correct dosages of unfractionated heparin (UFH), currently the most widely used anticoagulant for this purpose. Failure to adhere to correct dosages of UFH exposes patients to the risk of blood clotting in the extracorporeal circuit or prolonged time for arterio-venous fistulae cannulated sites to stop bleeding (Davenport 2009) at the conclusion of the session. Renal unit practitioners need specialised knowledge and skill pertaining to the use of UFH in haemodialysis. Surprisingly, there is a dearth of available current literature on renal unit practitioners’ knowledge and practice regarding the safe administration of UFH, and even less available literature on practitioners’ attitudes concerning safe administration of UFH.

### Aims of the study

The primary purpose of the study was to describe renal unit practitioners’ knowledge, attitudes and practice (KAP) regarding the safe use and effects of UFH in selected adult, chronic haemodialysis centres in the Cape Town Metropole, South Africa. The secondary purpose was to determine whether there was an association between selected independent variables (category of renal unit practitioner, years of experience, duration of orientation period and in-service education in pharmacological actions of UFH) and practitioners’ KAP concerning the safe use and effects of UFH. The null hypotheses stated that the independent variables did not influence practitioners’ KAP regarding safe use and effects of UFH.

### Background

UFH has been used since the inception of chronic haemodialysis therapy in the 1930s. For patients on chronic haemodialysis, a nephrologist prescribes treatment for about 3–4 hours two or three times a week. During the haemodialysis, 200–250 mL/minute of the patient‘s blood volume is extracorporeal and can clot, but to reduce the risk of clotting (Roy & Kalra 2012:107) an anticoagulant such as UFH is administered. In local renal settings the South African Renal Society guidelines (2006) and other international guidelines are provided on the use and dosage of UFH.

Although 184 related published studies between 2000 and 2014 were located in three databases, no KAP studies related to renal practitioners’ use of UFH appear to have been published. One systematic review by the Cochrane Renal Group (Palmer *et al.* 2014) was located but was unrelated to the present study. Renal unit practitioners are reported to be confused, concerned and even outspoken about safety aspects of UFH when preparing, administering and monitoring its effects (Pittard 2001:75; Baglin *et al*. 2006:21; Brunet *et al*. 2008:794). Suranyi and Chow (2010:386) have appealed to nurses to regularly review and update their knowledge regarding UFH.

### Trends

UFH prevents clotting in the extracorporeal haemodialysis circuit, but decades after its first use warnings continue of it being a high-alert medication (Institute for Safe Medication Practices [ISMP] 2006a) If safety precautions such as ensuring the right dose of UFH at the right time of administration and close monitoring are absent during haemodialysis therapy, serious adverse consequences such as anaphylaxis, heparin-induced thrombocytopenia and vascular access bleeding can result (Pittard 2001:75; Furushahi *et al*. 2002:1457; Bircher *et al.* 2006:1437). In the United Kingdom (UK), the National Patient Safety Agency (NPSA 2006) reported that anticoagulants are often the cause of preventable harm to patients, which can result in admission to hospital and even death. The Joint Commission on Accreditation of Healthcare Organisations (JCAHO) in the United States of America (USA) developed a project supporting the adoption of safety measures for anticoagulants as a National Patient Safety Goal, implemented in January 2009 (JCAHO 2009).

Approximately 35% of UFH is filtered by normal kidneys, but for patients on chronic haemodialysis, excess heparin cannot be excreted as it cannot be dialysed (Pittard 2001:75). Most protocols for use of UFH in the participating Cape Town Metropole dialysis centres prescribe a standard dose of 5000 IU of heparin three times a week for patients during haemodialysis sessions. This means that a patient's yearly dose of UFH amounts to approximately 780 000 IU, thus, predisposing the patient to long-term effects of UFH, such as osteoporosis (Pittard 2001:75; Furuhashi *et al.* 2002:1457). Although anticoagulants are widely used, errors and lack of tight control of the treatment of patients receiving anticoagulants continue (Institute for Healthcare Improvement [IHI] 2008) and heparin ‘is frequently unmonitored, untested and unsupervised in many dialysis facilities’ (Pittard 2001:75).

The NPSA reported 480 cases of patient harm from the use of anticoagulation therapy in the UK between 1990 and 2002 (NPSA 2006), of which 28 deaths were associated with the use of UFH but not linked to deaths of patients on chronic haemodialysis. Errors related to UFH accounted for 2.1% of total records submitted to the USA MedMarx national error database, of which 4.5% – 5.5% were harmful (Niccolai *et al.* 2004:146S). In a USA study (Grissinger *et al*. 2010:195), patient harm from UFH accounted for 1.4% to 4.9% of the reports submitted, occurring mostly (56%) during administration. Policy guidelines on monitoring baseline clotting studies before the administration of the initial and subsequent doses of UFH were available in only one of the seven Cape Town dialysis centres included in the study. In the other six centres, nephrologists prescribed individual patient dosages of UFH on medication charts. Five of the seven dialysis centres had a protocol on how to administer UFH. No data on adverse events (AEs) or deaths of adult patients on chronic haemodialysis related to UFH were available from the Cape Town Metropole. Moreover, there is no published data on morbidity and mortality associated with anticoagulation therapy within the South African renal population.

The results of this local study fill a gap in existing published data on renal practitioners’ KAP concerning the safe use of UFH, which may contribute to some extent to evidence-based renal nursing practice in South Africa to advance quality care and patient safety.

### Definition of key concepts

Adverse event: a negative situation arising from medical care that results in patient injury from unsafe care rendered, whether intentional or unintentional (World Health Organisation 2005).
Anticoagulant: an agent that stops blood from clotting (Dorland 2007:103).
Chronic kidney disease / end-stage kidney failure (synonym): failed natural kidney functioning. Kidneys are unable to filter toxins and maintain fluid balance.
Chronic haemodialysis centre / unit: treatment centre where patients with ESKF receive chronic haemodialysis therapy.
Clinical technologist: has a 3–year diploma or 4–year bachelor's degree in nephrology clinical technology, who is registered with the Health Professions Council of South Africa and who is permitted to perform acute and chronic adult haemodialysis, paediatric haemodialysis and peritoneal dialysis, and also monitor haemodialysis water treatment plants, and procedures in nephrology, including patients’ vital signs monitoring, intravenous cannulation and administration, administering blood transfusions, and administering drugs and managing their side-effects (South African Qualifications Authority 2014).
Enrolled (staff) nurse: practises basic nursing in the manner and to the level prescribed under the supervision of a professional nurse (South African Nursing Council, Republic of South Africa 2005:ch2).
Registered (professional) nurse: is qualified and competent to independently practise comprehensive nursing in the manner and to the level prescribed, and who is capable of assuming responsibility and accountability for such practice (South African Nursing Council, Republic of South Africa 2005:ch2).
Renal unit practitioner: registered nurses (RNs), enrolled nurses (ENs) and clinical technologists (CTs) working in a haemodialysis centre.
Unfractionated heparin / heparin: a ‘commercially’ prepared drug (Niccolai *et al*. 2004:146S) that indirectly inhibits the clotting cascade of events and prevents the formation of blood clots (Fischer 2007:181).

### Contribution to field

The study addresses gaps in the published literature concerning UFH prescription practices, dosage, administra­tion and monitoring of its effects (Ouseph & Ward 2000:181; Pittard 2001:75; Baglin *et al.* 2006:21; Brunet *et al.* 2008:794).

## Ethical considerations

Principles of the Helsinki Declaration (World Medical Association 2008) were upheld. After ethics approval (FHS HREC Ref.642/2012) of the protocol by the University of Cape Town (UCT) Faculty of Health Sciences’ Human Research Ethics Committee, institutional gatekeepers gave permission to gain entry to the research sites. The informed consent form assured potential research respondents that participation was voluntary, withdrawal from the study at any time would incur no penalties, encryption ensured their anonymity and information they provided was confidential.

There were no anticipated hazards and none were reported. A potential benefit of this study is to provide data from a South African perspective. These data may be useful for improved service delivery and in-service training programmes to promote patient safety.

## Research method and design

### Design

A descriptive cross-sectional design was employed for a KAP survey amongst renal unit practitioners (RNs, ENs and CTs) between March and April 2013.

### Setting

Seven adult dialysis centres (Table 1), from two tertiary government hospitals and five dialysis centres from a private dialysis provider, were invited to participate.

**TABLE 1:** Dialysis units included in the study.

Information categories	Dialysis institutions/Administration body						
	Public		Private				
	Centre 1	Centre 2	Centre 3	Centre 4	Centre 5	Centre 6	Centre 7
Number of dialysis units	1	1	1	1	1	1	1
Classification	Tertiary	Tertiary	n/a	n/a	n/a	n/a	n/a
Registered nurses *n* (%)	8 (38.1%)	17 58.6)	8 (66.7)	10 (76.9)	14 (73.7)	4 (100)	5 (83.3)
Enrolled nurses *n* (%)	5 (23.8%)	5 (17.2)	1 (8.3)	1 (7.7)	2 (10.5)	-	1 (16.7)
Clinical technologists *n* (%)	8 (38.1)	7 (24.1)	3 (25)	2 (15.4)	3 (15.8)	-	-
**Total number of permanent renal unit practitioners (*N* = 104)**	**21**	**29**	**12**	**13**	**19**	**4**	**6**

Number of dialysis units included in study: *n* = 7. n/a, not applicable.

### Sampling

A purposive sampling method was employed. To estimate a sample size in the absence of published data, the prevalence of a satisfactory level of knowledge of the safe use and effects of UFH amongst respondents was used and set at 50% (*n* = 52) of the population (*N* = 104), guided by personal clinical experience. Using StatCalc (EpiInfo Version 7), a sample size of 82 was determined. Although attitudes and practice scores were also investigated, these variables were not used to estimate a sample size.

## Instrumentation

A self-administered questionnaire was constructed, guided by the study aims and objectives, personal experience and available published literature, to ensure content validity. The development stage of determining content validity incorporated three steps:

- domain identification (knowledge, attitudes and practice)
- item generation
- instrument formation (development of the survey questionnaire and deciding on a suitable sequence of the items) (Schilling *et al.* 2007:362).

The structured part of the questionnaire consisted of fixed, closed questions with pre-coded response choices for ease of counting answers for quantitative data and analysis (Bowling & Ebrahim 2007:405). Open-ended questions (Appendix 1 Table 1 - A1) minimised successful guessing to determine respondents’ actual KAP regarding UFH, but data are not presented in this article.

## Validity

Face and content validity of the questionnaire were determined by three experts using the Index of Content Validity (CVI) approach (Schilling *et al.* 2007). Overall, the experts’ evaluation of the importance of each question was positive, as most responses (44/45, 97.8%) fell in the ‘extremely relevant’ (4/4) category.

## Reliability

The questionnaire was then pilot tested for test-retest reliability by three different respondents, representing the same composition as those in the actual study but not participating in the main study, after an interval of one week (Marx *et al*. 2003:730). Test-retest data were unchanged for the RN, but for both the EN and CT, the second attempt was worse than the first. A Pearson product-moment correlation (which does not penalise systematic error) showed that for the RN there was a strong, positive correlation between test 1 and test 2 results, which was statistically significant (*r* = 1, *n* = 3, *P =* < 0.001). For the EN and CT there was also a strong positive correlation between test results, but this was not statistically significant. For the RN, systematic error could be attributed to a learning effect (Rousson, Gasser & Seifert 2002). For the EN and CT, a worse test-retest result could be attributed to a fatigue effect, which shows that the systematic error should be treated differently, according to the situation (Rousson *et al*. 2002). Neither learning effect nor fatigue effect are directly related to reliability.

## Data collection procedure

Unit managers agreed to recruit respondents who met inclusion criteria, obtained written consent, and distributed and collected completed, self-administered questionnaires within two weeks.

## Data protection and data analysis

Data from 36 (of 45) closed-ended questions were captured on password-protected pre-coded Microsoft® Office Excel® 2007 spreadsheets. Missing data were labelled ‘1001’ for descriptive and frequency statistics, but removed for inferential statistics and changed to a blank space to reduce skewed interpretations.

For quality assurance of data entry, a Masters-prepared professional nurse entered data from a random selection of 8 of 77 completed questionnaires (10.4%) onto a Microsoft® Office Excel® 2007 spreadsheet. There was a 93.3% (42/45) agreement and 6.7% (3/45) disagreement between recordings so accepted, and this was not too far off the predetermined level of acceptance set at 95%. Incorrect entries on the spreadsheets were corrected for two respondents.

Descriptive statistical analysis was performed for frequency distributions and measurement of central tendency (for continuous data) for demographic and KAP data. To analyse the categorical variables, cross-tabulation was performed using the Fisher's exact test to determine whether there was an association between selected independent variables and KAP concerning the use and effects of UFH.

Application of the logistic regression model tested the strength of association between the independent variables and KAP variables, expressed as an odds ratio (OR). A *P-*value of ≤ 0.05 at a 95% Confidence Interval (CI) denoted an association that was statistically significant.

As a preamble to the KAP results, the questionnaire response rate and respondents’ demographic characteristics are presented.

## Results

### Response rate

All recruited dialysis centres participated in the study (Table 1): two tertiary government hospital dialysis centres (28.6%) and five dialysis centres (71.4%) from a private dialysis provider. Of the total population (*N* = 104) of renal unit practitioners who met inclusion criteria, 77 (74.0%) participated in the study compared to the estimated sample size (*n* = 82, 93.9%), therefore, no questionnaires were excluded from the final data analysis, even those with missing data. The highest proportion of missing data (8.2%) was recorded for the knowledge section. There was a 70.0% response rate (35/50) of practitioners from tertiary hospitals, and from the private sector, it was 77.8% (42/54).

### Demographic characteristics

The majority of respondents (94.8%, 73/77) were South African citizens (Table 2). There was a statistically significant association (*P* = 0.002, SD±10.8) between the variable professional category and age. A Scheffé post-hoc test showed that the difference in age between RNs and CTs reached statistical significance (*P* < 0.001, 95% CI 7.56–19.41) and between ENs and CTs (*P* = 0.020, 95% CI 1.70–24.24), but not between RNs and ENs. The association between gender and professional category reached statistical significance (*P* < 0.001). There was a statistically significant association (*P* < 0.001) between the variable professional category and qualification. Most respondents (53.2%, 41/77) had 0–5 years of experience (mean 9.4, SD ± 9.3), which did not reach statistical significance for the professional category. There was a statistically significant association (*P* = 0.001) between the variable professional category and having or not having in-service education on the pharmacological actions of UFH.

**TABLE 2:** Demographics of respondents.

Variables	Professional Category		
	RN - *n* = 51	CT - *n* = 21	EN - *n* = 5
	*n* (%)	*n* (%)	*n* (%)
Age (*n* = 74*)			
21-30	5 (9.8)	10 (47.6)	0 (0.0)
31-40	10 (19.6)	8 (38.1)	1 (20.0)
41-50	19 (37.3)	2 (9.5)	4 (80.0)
51-60	12 (23.5)	1 (4.8)	0 (0.0)
> 61	2 (3.9)	0 (0.0)	0 (0.0)
Missing	3 (5.9)	0 (0.0)	0 (0.0)
Subtotal	48 (94.1)	21 (100.0)	5 (100.0)
Total	51 (100.0)	21 (100.0)	5 (100.0)
**Gender (*n* = 77)**			
Male	6 (11.8)	13 (61.9)	0 (0.0)
Female	45 (88.2)	8 (38.1)	5 (100.0)
Total	51 (100.0)	21 (100.0)	5 (100)
Citizen (*n* = 76*)			
South Africa	48 (94.1)	20 (95.2)	5 (100.0)
Other	2 (3.9)	1 (4.8)	0 (0.0)
Missing	1 (2.0)	0 (0.0)	0 (0.0)
Subtotal	50 (98.0)	21 (100.0)	5 (100.0)
Total	51 (100.0)	21 (100.0)	5 (100.0)
**Qualification (*n* = 77)**			
PGDNN	7 (13.7)	0 (0.0)	0 (0.0)
Dip CC	8 (15.7)	0 (0.0)	0 (0.0)
Dip Nephro CT	0 (0.0)	11 (52.4)	0 (0.0)
B Tech Nephro CT	0 (0.0)	8 (38.1)	0 (0.0)
OJT	27 (52.9)	0 (0.0)	5 (100.0)
PGDNN+Dip CC	2 (3.9)	0 (0.0)	0 (0.0)
Dip CC+OJT	6 (11.8)	0 (0.0)	0 (0.0)
Dip Nephro CT + B Tech Nephro CT	0 (0.0)	1 (4.8)	0 (0.0)
Dip Nephro CT + OJT	0 (0.0)	1 (4.8)	0 (0.0)
PGDNN+Dip CC + OJT	1 (2.0)	0 (0.0)	0 (0.0)
Total	51 (100.0)	21 (100.0)	5 (100.0)
**Experience in years (*n* = 77)**			
0-5	29 (56.9)	10 (47.6)	2 (40.0)
6-40	22 (43.1)	11 (52.4)	3 (60.0)
Total	51 (100.0)	21 (100.0)	5 (100.0)
Orientation by mentor (*n* = 76*)			
Yes	35 (68.6)	17 (81.0)	2 (40.0)
No	16 (31.4)	4 (19.0)	2 (40.0)
Missing	0 (0.0)	0 (0.0)	1 (20.0)
Subtotal	51 (100.0)	21 (100.0)	4 (80.0)
Total	51 (100.0)	21 (100.0)	5 (100.0)
**Duration of orientation (*n* = 77)**			
On orientation	1 (2.0)	0 (0.0)	0 (0.0)
Days	0 (0.0)	1 (4.8)	0 (0.0)
Weeks	14 (27.5)	6 (28.6)	5 (100.0)
Months	22 (43.1)	8 (38.1)	0 (0.0)
Not Applicable	14 (27.5)	6 (28.6)	0 (0.0)
Total	51 (100.1)	21 (100.1)	5 (100.0)
Inservice of UFH (*n* = 76*)			
Yes	13 (25.5)	11 (52.4)	5 (100.0)
No	37 (72.5)	10 (47.6)	0 (0.0)
Missing	1 (2.0)	0 (0.0)	0 (0.0)
Subtotal	50 (98.0)	21 (100.0)	5 (100.0)
Total	51 (100.0)	21 (100.0)	5 (100.00

*, denotes missing data.

BTech Nephro CT, bachelor in technology (nephrology); CT=clinical technologist; Dip CC, diploma in critical care; Dip Nephro CT, diploma in clinical technology nephrology); EN, enrolled nurse; OJT, on job training; PGDNN, postgraduate diploma in nephrology nursing; RN, registered nurse; UFH, unfractionated heparin.

### Respondents’ overall test scores for KAP

Respondents’ overall test scores achieved for KAP are presented in Table 3.

**TABLE 3:** Overall KAP scores.

KAP scores	Number of respondents	Minimum score	Maximum score	Mean score	SD
Knowledge scores (out of 18)	77	4	18	10.8	3.1
Attitudes scores (out of 41)	77	0	35	25.9	6
Practice scores (out of 50)	77	19	44	35.8	6

KAP, knowledge, attitudes and practice; SD, standard deviation.

The mean score for knowledge was 10.8/18 (60.0%); for attitude, 25.9/41 (63.2%); and for practice, 35.8/50 (71.6%).

### Knowledge

Results for specific questions on respondents’ knowledge of the use and effects of UFH are presented in Appendix 1 Table 1 - A1.

### Association between knowledge and selected independent variables

The association between knowledge and selected independent variables is presented in Table 4.

**TABLE 4:** Association between knowledge and selected independent variables.

Independent Variables	Knowledge		Fisher's exact test *P*-value
	Acceptable number (%)	Poor number (%)	
**Professional category (*n* = 77)**			
RN (*n* = 51)	42 (72.4)	9 (47.4)	0.011
CT (*n* = 21)	15 (25.9)	6 (31.6)	
EN (*n* = 5)	1 (20.0)	4 (80.0)	
Experience in years (*n* = 77*)			
0-5 (*n* = 41)	32 (55.2)	9 (47.4)	0.604
6-40 (*n* = 36)	26 (44.8)	10 (52.7)	
**Duration of orientation (*n* = 77)**			
On orientation (*n* = 1)	0 (0.0)	1 (5.3)	0.549
Days (*n* = 1)	1 (1.7)	0 (0.0)	
Weeks (*n* = 2 5)	20 (34.5)	5 (26.3)	
Months (*n* = 30)	22 (37.9)	8 (42.1)	
Not applicable (*n* = 20)	15 (25.9)	5 (26.3)	
**In-service education of UFH (*n*** **= 76)**			
Yes (*n* = 29)	19 (32.8)	10 (55.6)	0.101
No (*n* = 47)	39 (67.2)	8 (44.4)	

CT, clinical technologist; EN, enrolled nurse; RN, registered nurse; UFH, unfractionated heparin.

*, Adjustment of years of experience by knowledge both as continuous variables gave a Spearman's rho correlation of 0.571.

The data in Table 4 shows that overall, 75.3% (58/77) of respondents had an acceptable level of knowledge regarding UFH, whilst 24.6% (19/77) had poor knowledge. Most of the RNs (82.3%, 42/51) had an acceptable level of knowledge. The majority (80.0%, 4/5) of ENs had poor knowledge of UFH, despite the fact that as employees within haemodialysis units, they administer UFH under supervision of RNs. There was a statistically significant association (*P* = 0.011) between the variable professional category and self-reported knowledge of UFH. Bivariate logistic regression analysis showed that the odds of the ENs having poor knowledge regarding UFH compared to the RNs were 18.7 times higher at a 95% CI (1.9–187.4), and that this difference reached statistical significance (*P* = 0.013). The odds of the CTs having poorer knowledge than the RNs were 1.9 times higher, at a 95% CI (0.6–6.1), but this difference did not reach statistical significance (*P* = 0.304).

The majority of respondents 41.5% (32/77) who had an acceptable level of knowledge of UFH had 0–5 years of experience. Ten of the 77 respondents (12.9%) who had 11–35 years of experience displayed poor knowledge of UFH. There was no statistically significant association between the level of knowledge and the variables:

- years of experience (*P* = 0.604)
- duration of orientation (*P* = 0.549)
- in-service education (*P* = 0.101).

For in-service education on UFH, 1.3% (1/77) of respondents were not included in the calculation.

### Attitude

Data showed that the respondents’ lack of knowledge concerning the use and administration of UFH was reflected in their attitude to its use and administration. Data in Table 5 shows the association between attitudes and selected independent variables.

**TABLE 5:** Association between attitude and selected independent variables.

Independent Variables	Attitude		Fisher's exact test *P*-value
	Positive number (%)	Negative number (%)	
**Professional category (*n* = 77)**			
RN (*n* = 51)	38 (69.1)	13 (59.1)	0.637
CT (*n* = 21)	14 (25.5)	7 (31.8)	
EN (*n* = 5)	3 (5.5)	2 (9.1)	
Experience in years* (*n* = 77)			
0-5 (*n* = 41)	27 (49.1)	14 (63.6)	0.315
6-40 (*n* = 36)	28 (50.9)	8 (36.4)	
**Duration of orientation (*n* = 77)**			
On orientation (*n* = 1)	0 (0.0)	1 (4.5)	0.119
Days (*n* = 1)	0 (0.0)	1 (4.5)	
Weeks (*n* = 25)	17 (30.9)	8 (36.4)	
Months (*n* = 30)	21 (38.2)	9 (40.9)	
Not Applicable (*n* = 20)	17 (30.9)	3 (13.6)	
**In-service education on UFH (*n* = 76)**			
Yes (*n* = 29)	20 (37.0)	9 (40.9)	0.798
No (*n* = 47)	34 (63.0)	13 (59.1)	

CT, clinical technologist; EN, enrolled nurse; RN, registered nurse; UFH, unfractionated heparin.

*, Adjustment of years of experience by attitude, both as continuous variables gave a Spearman's rho correlation of 0.254.

Data in Table 5 show that most RNs (74.5%; 38/51), CTs (66.6%; 14/21) and ENs (60.0%; 3/5) had a positive attitude, but the association between professional category and attitude did not reach statistical significance. The association between years of experience and attitude and between in-service education on UFH and attitude was not statistically significant.

### Practice

Results for specific questions on respondents' practice regarding the use and effects of UFH are not presented in this article, but are available on request from the authors. Data in Table 6 show the association between practice and selected independent variables.

**TABLE 6:** Association between practice and selected independent variables.

Independent Variables	Practice		Fisher's exact test *P*-value
	Acceptable number (%)	Unacceptable number (%)	
**Professional category (*n* = 77)**			
RN (*n* = 51)	38 (65.5)	13 (68.4)	0.517
CT (*n* = 21)	15 (25.9)	6 (31.6)	
EN (*n* = 5)	5 (8.6)	0 (0.0)	
Experience in years (*n* = 77*)			
0-5 (*n* = 41)	26 (44.8)	15 (78.9)	0.016
6-40 (*n* = 36)	32 (55.2)	4 (21.1)	
**Duration of orientation (*n* = 77)**			
On orientation (*n* = 1)	0 (0.0)	1 (5.3)	0.601
Days (*n* = 1)	1 (1.7)	0 (0.0)	
Weeks (*n* = 25)	19 (32.8)	6 (31.6)	
Months (*n* = 30)	23 (39.7)	7 (36.8)	
Not applicable (*n* = 20)	15 (25.9)	5 (26.3)	
**In-service education of UFH (*n* = 76)**			
Yes (*n* = 29)	26 (45.6)	3 (15.8)	0.028
No (*n* = 47)	31 (54.4)	16 (84.2)	

CT, clinical technologist; EN, enrolled nurse; RN, registered nurse; UFH, unfractionated heparin.

*, Adjustment of years of experience by practice, both as continuous variables gave a Spearman's rho correlation of 0.009.

Across the different professional categories of renal unit practitioners, the results reveal that most had an acceptable level of self-reported competency regarding the use of UFH. The association between the independent variable professional category and the dependent variable practice was not statistically significant (*P* = 0.517).

Of the respondents with 0–5 years’ experience, most (63.4%, 26/41) reported an acceptable level of practice competency. The association between years of experience and self-reported practice concerning UFH was statistically significant (*P* = 0.016), therefore, bivariate logistic regression analysis was performed for strength of association between the two variables and for statistical significance, before making further judgements about the null hypothesis. Respondents who had 0–5 years of experience were 4.6 times more likely to report having poor practice at a 95% CI (1.4–15.6), than respondents who had more than six years of experience, and this difference reached statistical significance (*P* = 0.014).

There was no statistically significant association between duration of orientation and self-reported good practice (15/20, 75.0%) in relation to UFH. The results of the Fisher's exact chi-squared test revealed that the association between in-service education and practice reached statistical significance (*P =* 0.028), and bivariate logistic regression analysis was performed. The odds of respondents who had no in-service education reporting poor practice were 4.3 times higher, at 95% CI (1.1–16.5), than respondents who had education in pharmacological actions of UFH, and this difference reached statistical significance (*P* = 0.032).

The outcomes of the logistic regression tests confirm rejection of the null hypotheses that the variables professional category, years of experience and in-service education on the pharmacological actions of UFH do not influence renal unit practitioners’ KAP. However, the null hypothesis, that the variable duration of orientation did not affect the renal unit practitioners’ KAP, was accepted.

## Discussion

Chronic haemodialysis treatment is the most common renal replacement therapy for patients diagnosed with Stage 5 adult ESKF. The patient is at risk of bleeding or clotting if the administered individualised dose of UFH is not appropriate. In the USA there is no standard heparin dose for patients on chronic haemodialysis and little is known about safety aspects of UFH in this context (Shen & Winkelmayer 2012:473). However, international health organisations (NPSA 2006; ISMP 2006b; IHI 2008; Agency for Healthcare Research and Quality [AHRQ n.d.]) have appealed for safer practice to minimise avoidable complications experienced by patients receiving UFH.

The primary purpose of the study was achieved by describing renal practitioners’ KAP regarding the use of UFH in selected adult chronic haemodialysis centres. Published medication KAP studies were located but not specific to UFH. Studies on nurses’ knowledge of pharmacology conducted in the UK (Ndosi & Newell 2008) and in Taiwan on nurses’ knowledge of high-alert medication (Hsaio *et al*. 2010) recommend that, to be competent in medication administration, nurses should strive continuously to update their medication knowledge with supplementary pharmacology education programmes.

The secondary aim was achieved by establishing whether or not there was an association between selected independent variables and renal practitioners’ KAP. The aims of the study were achieved by objectives.

## Outline of the results compared to the published literature

Most respondents were in the middle adulthood age group (40–65 years) which, according to Erik Erikson's 1959 theory, is stage seven of psychosocial development, implying that if their needs are unfulfilled, they may experience ‘stagnation’ and feel ‘unproductive’ (McLeod 2008:3). In haemodialysis centres it is good practice (Community Tool Box 2013:2) for new staff to be assigned to a mentor for orientation before taking responsibility for patient care. Although this did occur, for most respondents the orientation did not include in-service education in UFH.

### Respondents’ self-reported knowledge of UFH

Results of a Malaysian KAP study on general medication amongst 40 medical nurses showed adequate knowledge (mean scores: knowledge 13.8, attitude 16.4 and practice 10.7), but a lack of in-depth knowledge of aspects of pharmacology (Raja, Daud & Syed 2009:17). The mean score for nursing students’ knowledge of pharmacology in 29 nursing schools was 55% and for medication calculation skills 66%, but they performed poorly in knowledge retention and practical implementation in clinical areas (Dilles *et al.* 2011:499). In our study the majority of RNs and CTs had an acceptable level of knowledge of UFH but, overall, respondents reported incorrectly that UFH breaks down blood clots, whereas it prevents blood clots forming (Fischer 2007:178; Lankshear, Harden & Simms 2010:49), as UFH is not a fibrinolytic agent. Most respondents did not know the correct antidote to or dosage for managing UFH overdose (1 mg of protamine sulphate to neutralise 100 IU of UFH [European Best Practice Guidelines Expert Group on Haemodialysis 2002:65; Baglin *et al*. 2006:28; Lankshear *et al.* 2010:51; Lemon & Crannage 2011:212]). Most respondents did not know the reason for not administering UFH orally (it is poorly absorbed). Most respondents did not know that the type of heparin used in their unit was a porcine derivative which carries a greater risk of anaphylaxis (Shen & Winkelmayer 2012:475)

Some respondents’ inability to calculate correct dosages of UFH was of deep concern, as this can have life-threatening consequences for patients. Nurses’ medicating deficiencies were highlighted in a study conducted on medication knowledge, dose calculation (Barkhouse-Mackeen & Murphy 2013:91) and medication errors which resulted from poor calculation skills (Polifroni, McNulty & Allchin 2003:458; Wright 2007:279). A study on numerical skills and drug calculations in relation to patient safety, conducted in the UK in 2006 (McMullan, Jones & Lea 2010:891) showed, that 55% (126/229) of nursing students and 45% (20/44) of RNs failed the numeracy test, and 92% (211/229) of the students and 89% (39/44) of RNs failed the calculation test.

### Respondents’ self-reported attitudes regarding the use and effects of UFH

Respondents’ lack of knowledge in certain areas was reflected in their attitude to the use and administration of UFH, but extrapolation to mean that poor knowledge translates to having poor attitudes must be interpreted with caution. Only a few respondents were greatly concerned that patients may experience adverse effects of UFH, implying a casual attitude regarding such a high-alert medication. More than half of the respondents felt uncomfortable administering UFH that had been prepared by others, and this attitude to practice is supported in the published literature (Chevalier *et al*. 2011:344).

The majority of respondents displayed a positive attitude in relation to the administration of UFH. Respondents with 0–5 years of experience (65.9%; 27/41) presented the most positive attitude.

### Respondents’ self-reported practice regarding the use and effects of UFH

The majority of all categories of renal practitioners reported having acceptable practice. The majority would always give the UFH dose prescribed by the nephrologist, and about half of them would contact the prescribing doctor if concerned about a patient's prescribed dose, whilst a few would adjust the dose and document the adjusted dose. Rather than first consulting the medicines formulary for UFH information, respondents would consult a doctor. Most respondents would always monitor patients for anaphylactic shock and few would never verify allergies with patients before administering UFH. The majority of respondents would always check the UFH dose with another person before administration, rather than only doing so sometimes. Most respondents would always document adverse effects of UFH and report related adverse effects in patients to unit managers. A study by Hanafi *et al*. (2012:21) in Tehran showed that 91% of nurses had never reported an adverse drug reaction (ADR) directly to the Iran Adverse Drug Reaction National Centre and 35% were unaware of the centre's existence, but were most likely to report to doctors (87.1%) and pharmacists (1.8%). The reasons for not reporting ADR were:

- uncertainty of the adverse reactions (49.5%)
- unawareness of reporting procedure (45.1%)
- lack of time to complete a report (33%) (Hanafi 2012:21).

However, Niccolai *et al*. (2004:148S) suggest that non-reporting directly to ADR databases could be attributed to nurses fearing reprisal and blame. A multinational systematic review of empirical evidence on the prevalence and nature of medication administration errors (Keers *et al*. 2013:1) showed that, despite improvement in medication safety, higher medication administration error (53.3%) occurred via the intravenous route compared to other routes.

Study findings suggest that currently, in the seven research sites, there is not a set schedule for coagulation studies for patients on haemodialysis. Pittard (2001:75) and Winkler *et al*. (2007:499) support the need for strict laboratory monitoring of coagulation studies of patients, to establish a therapeutic dose to prevent under or over-anticoagulation. Respondents did not consider prolonged bleeding or clotting of patients’ blood circuits (Pittard 2001:74) to be the adverse effects of UFH, which may account for them reportedly not having recently witnessed an adverse event. Respondents had not kept up to date with literature specifically on UFH, a concern echoed by Ndosi and Newel (2008:570) and Raja *et al*. (2009:17) that, in general, nurses lack in-depth knowledge of medication.

## Limitations of the study method

Limiting the search strategy to published studies on renal unit practitioners’ KAP regarding use and effects of UFH, limited the scope of the literature review. Had the search strategy included studies relating to KAP amongst health practitioners and to thromboprophylaxis more broadly, more studies might have been located. Structured individual interviews may have resulted in less missing data (total missing data = 169/3388 [5.0%]). Missing data should have been addressed during validation of the questionnaire and interpretation of the data.

The attitude section of the questionnaire was the most difficult to develop, as this is a subjective component, and it was measured using both Likert scale and closed-ended questions. Questions to elicit the respondents’ attitude were based on the respondents’ knowledge and practice regarding the use and effects of UFH, warranting cautious interpretation of responses. Post-hoc internal consistency of the questionnaire was performed, but this should have been before the experts validated the questionnaire.

There were problems with the pilot study sample size (*n* = 3 of an estimated 82, 4%) for testing the instrument reliability, as it fell short of an estimated pilot sample size of 10, recommended by Nieswiadomy (2002). Therefore, the validation results have to be interpreted with caution. Intra-class correlation (ICC) for testing reliability of the ratings for a typical, single rater (which does penalise systematic error) was not calculated, as there was only one rater within each group. The reliability testing of the instrument may have been strengthened, had Cohen's kappa or Fleiss kappa tests been performed, and also construct validity of the instrument.

The study did not achieve the estimated sample size of 82 (1.0% margin of error, 95% CI). Instead, 77/82 (93.9%) respondents participated and this has implications for informing the implementation of the results. Professional category as an independent variable should not have been included as a measure against dependent variables KAP, because of the unequal distribution of the different professional categories. In hindsight, perhaps the term dialysis personnel should have been used as an independent variable, without differentiating between the categories of professionals. Alternatively, the ENs could have been excluded as they do not have a specialist nephrology qualification.

The study would have been strengthened if a minimum acceptable knowledge score had been set, such as a more clinical criterion based on patient safety and what practitioners should know, benchmarked at the 75% quartile level.

### Strengths and evaluation of the study

This study was evaluated using The Strengthening the Reporting of Observational Studies in Epidemiology (STROBE) (Vandenbroucke *et al.* 2007) checklist, to ensure that all the important aspects of a descriptive cross-sectional study were attended to.

### Implications and recommendations for education

The limited statutory scope of practice for ENs (South African Nursing Council, Republic of South Africa 1984) was reflected in their poor knowledge of UFH, and this has implications for their placement in haemodialysis units. Therefore, ENs should not practise independently in a haemodialysis unit, but at all times under the supervision of a registered nurse and not a clinical technologist. Pre and post-registration nursing education programmes should emphasise medication calculation, pharmacokinetics, pharmacotherapeutics and pharmacodynamic aspects of UFH. Nurses should be taught how to navigate medicine formularies and how to do literature searches to improve their medication knowledge to ensure life-long learning.

### Implications for practice and recommendations for clinical management teams

Guidelines for safe administration of UFH and monitoring practices should be developed through consensus by an expert panel. Management teams should implement problem-based, in-service education programmes that include pharmacology, pharmacokinetics, pharmacodynamics, medication calculation and pharmacotherapeutics. Improving staff KAP regarding UFH should limit medical litigation and financial claims against individual health care personnel and institutions. Management teams should encourage renal unit practitioners to enrol for patient safety programmes such as the webinar by ISMP (2013:1), ‘Improving Medication Safety through Staff Education and Competency Assessment: An Important Challenge for Healthcare Organisations’.

Management teams should establish pharmaco-vigilance units to encourage reporting of incidents and unsafe practice without naming, blaming and shaming, to augment the renal database of patients who experienced AEs associated with UFH. Staff could be provided with a bedside calibrated point-of-care testing apparatus to measure patients’ activated partial thromboplastin time instead of having to wait for laboratory results.

Before new staff administer UFH, their competency level should be evaluated before and after the orientation programme and, if they do not pass the predetermined set score, remedial intervention should be instituted. Staff competency tests should be conducted annually and if results are poor, re-training and re-testing should be offered to ensure ongoing employment.

### Unanswered questions and recommendations for future research

Shen and Winkelmayer (2012:483) suggest that large cluster randomised trials should be conducted in haemodialysis units to confirm the safe therapeutic range for different anticoagulants or pertaining to their risks and benefits. A renal nursing practice model should be developed for the local South African context for both the public and private chronic haemodialysis centres.

## Conclusion

Results suggest that renal practitioners lack clinical guidelines for the safe use and monitoring of UFH. This has serious implications for patients receiving chronic haemodialysis and there are medico-legal consequences for individual health practitioners and their employers. Knowing that professional category influences knowledge and safe use of UFH, and that ENs had poor knowledge, their role in haemodialysis units needs urgent attention. Results suggest that there is a direct relationship between years of experience and quality of haemodialysis practice, and between having in-service education and quality of practice. Therefore, on-duty rosters should take these factors into consideration to ensure optimal patient outcomes.

## Appendix 1

**TABLE 1-A1:** Respondents’ self-reported knowledge results by professional category.

Questions and responses	Professional category (*n* = 77)		
	RN (*n* = 51) Number (%)	CT (*n* = 21) Number (%)	EN (*n* = 5) Number (%)
**Q010. Query bleeding tendencies before UFH is administered**			
Yes	51 (100.0)	19 (90.5)	5 (100.0)
No	0 (0.0)	1 (4.8)	0 (0.0)
Missing	0 (0.0)	1 (4.8)	0 (0.0)
Total	51 (100.0)	21 (100.0)	5 (100.0)
**Q011. UFH breaks down blood clots?**			
Yes	25 (49.0)	4 (19.0)	1 (20.0)
No	25 (49.0)	16 (76.2)	3 (60.0)
Unsure	0 (0.0)	1 (4.8)	1 (20.0)
Missing	1 (2.0)	0 (0.0)	0 (0.0)
Total	51 (100.0)	21 (100.0)	5 (100.0)
**Q012. Effects of administering UFH with low aPTT**			
Bleeding	22 (43.1)	11 (52.4)	3 (60.0)
Nil ill effects	12 (23.5)	1 (4.8)	0 (0.0)
Bleeding and unscheduled hospitalisation	16 (31.4)	8 (38.1)	0 (0.0)
Missing	1 (2.0)	1 (4.8)	2 (40.0)
Total	51 (100.0)	21 (100.0)	5 (100.0)
**Q013. Blood pressure reading of 240/140 mmHg. Administer UFH or not**			
Yes	5 (9.8)	1 (4.8)	1 (20.0)
No	42 (82.4)	17 (81.0)	3 (60.0)
Unsure	4 (7.8)	1 (4.8)	0 (0.0)
Missing	0 (0.0)	2 (9.5)	1 (20.0)
Total	51 (100.0)	21 (100.0)	5 (100.0)
**Q014. UFH not administered following platelet count**			
420-450 x 10^9/1^	4 (7.8)	0 (0.0)	0 (0.0)
50-80 x 10^9/1^	41 (80.4)	15 (71.4)	4 (80.0)
150-180 x 10^9/1^	4 (7.8)	3 (14.3)	0 (0.0)
Missing	2 (3.9)	3 (14.3)	1 (20.0)
Total	51 (100.0)	21 (100.0)	5 (100.0)
**Q015. Method to arrest bleeding post-haemodialysis session to counteract effects of UFH**			
PS 1 mg/100 IU UFH	23 (45.1)	5 (23.8)	1 (20.0)
PS 2 mg/1000 IU UFH	10 (19.6)	8 (38.1)	3 (60.0)
Vitamin K 1 mg	9 (17.6)	1 (4.8)	0 (0.0)
Unsure	9 (17.6)	6 (28.6)	0 (0.0)
Missing	0 (0.0)	1 (4.8)	1 (20.0)
Total	51 (100.0)	21 (100.0)	5 (100.0)
**Q016. Reason UFH not administered orally**			
Effectiveness	4 (7.8)	0 (0.0)	0 (0.0)
Effects	23 (45.1)	9 (42.9)	2 (40.0)
Pharmacokinetics	9 (17.6)	1 (4.8)	0 (0.0)
Destroyed by stomach acids	10 (19.6)	8 (38.1)	0 (0.0)
Unsure	1 (2.0)	1 (4.8)	0 (0.0)
Missing	4 (7.8)	2 (9.5)	3 (60.0)
Total	51 (100.0)	21 (100.0)	5 (100.0)
**Q017. Importance of having the patient's baseline urea values before administering the first dose of UFH**			
Bleeding risk	31 (60.8)	12 (57.1)	1 (20.0)
Increased urea levels	1 (2.0)	0 (0.0)	1 (20.0)
Risk of complications	4 (7.8)	3 (14.3)	0 (0.0)
Unsure	4 (7.8)	1 (4.8)	0 (0.0)
Dosage	1 (2.0)	0 (0.0)	0 (0.0)
Information	1 (2.0)	0 (0.0)	0.(0.0)
Missing	9 (17.6)	5 (23.8)	3 (60.0)
Total	51 (100.0)	21 (100.0)	5 (100.0)
**Q018. Route of administration of UFH**			
Intravenously	37 (72.5)	11 (52.3)	5 (100.0)
Via dialysis machine	13 (25.5)	7 (33.3)	3 (60.0)
Bolus dosage	1 (2.0)	2 (9.5)	0 (0.0)
Missing	0 (0.0)	1 (4.8)	**0 (0.0)**
Total	51 (100.0)	21 (100.0)	2 (40.0)
**Q019. Type of UFH used**			
Porcine	20 (39.2)	7 (33.3)	1 (20.0)
Bovine	10 (19.6)	6 (28.6)	0 (0.0)
Other	10 (19.6)	0 (0.0)	2 (40.0)
Unsure	8 (15.7)	6 (28.6)	0 (0.0)
Missing	3 (5.9)	2 (9.5)	2 (40.0)
Total	51 (100.0)	21 (100.0)	5 (100.0)
**Q020. UFH temperature storage**			
Patient temp	1 (2.0)	0 (0.0)	0 (0.0)
< 25 degrees C	32 (62.7)	18 (85.7)	2 (40.0)
> 25 degrees C	6 (11.8)	1 (4.8)	0 (0.0)
Room temp	6 (11.8)	1 (4.8)	2 (40.0)
Missing	6 (11.8)	1 (4.8)	1 (20.0)
Total	51 (100.0)	21 (100.0)	5 (100.0)
**Q021. A patient needs to receive 2 000 IU in stock 20 000 IU of UFH. Calculate the dose of UFH**			
Hyperkalemia	22 (43.1)	8 (38.1)	2 (40.0)
Nil	9 (17.6)	1 (4.8)	2 (40.0)
Don’t know	8 (15.7)	5 (23.8)	0 (0.0)
Inhibit the secretion of aldosterone-hyperkalemia	3 (5.9)	2 (9.5)	0 (0.0)
Missing	9 (17.6)	5 (23.8)	1 (20.0)
Total	51 (100.0)	21 (100.0)	5 (100.0)
**Q22. A patient needs to receive 2 000 IU in stock 20 000 IU of UFH. Calculate the dose of UFH**			
10 mL	32 (62.7)	15 (71.4)	0 (0.0)
2000 IU	0 (0.0)	2 (9.5)	0 (0.0)
10 ML- 100 IU	0 (0.0)	1 (4.8)	0 (0.0)
01-5 mL	5 (9.8)	0 (0.0)	0 (0.0)
10-100	5 (9.8)	0 (0.0)	1 (20.0)
1000 IU	3 (5.9)	0 (0.0)	0 (0.0)
10 000 IU	1 (2.0)	0 (0.0)	0 (0.0)
Formula only	2 (3.9)	0 (0.0)	1 (20.0)
Missing	3 (5.9)	3 (14.3)	3 (60.0)
Total	51 (100.0)	21 (100.0)	5 (100.0)
